# Plasma amino acid profiles in pediatric obesity: potential biomarkers for the early assessment of metabolic risk

**DOI:** 10.3389/fped.2025.1631302

**Published:** 2025-09-26

**Authors:** Ece Öge Enver, Aydın Duygu, Pınar Vatansever, Bilal Yılmaz, Yasemin Akın

**Affiliations:** ^1^ Division of Pediatric Nutrition and Metabolic Diseases, Department of Pediatrics, Kartal Dr. Lütfi Kırdar City Hospital, Istanbul, Türkiye; ^2^ Amino Acid Science Clinics, London, United Kingdom; ^3^ Department of Biochemistry, Kartal Dr. Lütfi Kırdar City Hospital, Istanbul, Türkiye; ^4^ Department of Pediatrics, Kartal Dr. Lütfi Kırdar City Hospital, Istanbul, Türkiye

**Keywords:** pediatric obesity, branched-chain amino acids (BCAAs), metabolic risk, amino acid profiling, insulin resistance

## Abstract

**Introduction:**

Childhood obesity is among the most serious and rapidly growing public health issues globally. Although body mass index (BMI) is commonly used to evaluate obesity, it does not always reflect early metabolic disturbances. Recent studies have emphasized the importance of metabolomics, particularly plasma amino acid profiling, in detecting subclinical metabolic risk. In this context, branched-chain amino acids (BCAAs) have emerged as potential early biomarkers of insulin resistance and cardiometabolic risk.

**Methods:**

This cross-sectional study included 97 participants aged 5–18 years, including 56 children with obesity (BMI ≥ 95th percentile) and 41 healthy controls. Anthropometric measurements, as well as fasting glucose, insulin, lipid profile, and HbA1c levels, were recorded. Plasma concentrations of 44 amino acids were measured using liquid chromatography–tandem mass spectrometry (LC–MS/MS) with a commercial kit (JASEM®, Agilent Ultivo Triple Quadrupole LC–MS). BCAA levels and relevant ratios, such as glycine/BCAA and glutamic acid/glutamine, were calculated. Receiver operating characteristic (ROC) curve analyses were performed to evaluate the diagnostic performance of key variables.

**Results:**

Children with obesity had significantly higher levels of BCAAs and other amino acids, including phenylalanine, tyrosine, alanine, and glutamic acid (all *p* < 0.05). Conversely, glycine, serine, and asparagine levels were significantly lower in children with obesity. Fasting insulin emerged as a strong predictor of obesity [area under the ROC curve (AUC) = 0.87], while total BCAAs also displayed strong predictive performance (AUC = 0.78). A reduced glycine/BCAA ratio and an increased glutamic acid/glutamine ratio were associated with early metabolic dysregulation.

**Conclusion:**

Our findings highlight the potential of plasma amino acid profiling as a supportive tool for the early assessment of metabolic risk in children with obesity. The integration of amino acid-based indices could improve risk classification and support personalized preventive strategies in pediatric populations.

## 1 Introduction

The incidence of childhood obesity, an important public health problem, has rapidly increased globally in recent years, forming the basis of metabolic diseases in adulthood. The World Health Organization reports that approximately 18% of children aged 5–19 years worldwide are overweight, reflecting an almost 10-fold increase over the last four decades (1). Excess weight and obesity in early childhood increase the risk of these conditions in adulthood, thereby elevating the risks of chronic inflammatory diseases such as type 2 diabetes, cardiovascular disease, non-alcoholic fatty liver disease, and certain cancers (2, 3).

There is growing evidence that childhood obesity is associated with both energy imbalance and metabolic disorders at the cellular level. In this context, body mass index (BMI) or fasting insulin levels alone are insufficient to identify the metabolic risk of children with obesity. Although some children can be metabolically healthy despite high BMI, others could be in a “silent” phase of metabolic risk before insulin resistance develops. The use of new biomarkers is of great importance for identifying children at risk. Therefore, metabolomic analysis is critical for screening and intervening in childhood obesity (4, 5). Recently, investigations of early screening and diagnosis using metabolomics have been conducted (6).

Nutrients, such as glucose, amino acids, and lipids, influence each other; nevertheless, amino acids are critical modifiers in obesity. As amino acids reflect internal metabolic changes and the intestinal microbiota and act as metabolic modulators, metabolomic analysis might be useful for monitoring amino acid levels and predicting metabolic disorders during childhood (7). Recent studies have suggested that branched-chain amino acids (BCAAs; valine, leucine, and isoleucine) play key roles in obesity-related metabolic disorders. Several metabolomic studies found that BCAA levels are markedly elevated in children and adults with obesity, and this increase is closely associated with the development of insulin resistance, glucose intolerance, and type 2 diabetes (8, 9). BCAAs are believed to reduce insulin sensitivity by affecting mTOR signaling pathways in liver and muscle cells and trigger inflammatory processes by disrupting mitochondrial energy metabolism (10).

Furthermore, the plasma amino acid profile can be used to predict both the current biochemical effects of obesity and prospective cardiometabolic risk. In this context, amino acid analyses could provide early indications of the metabolic burden in childhood before clinical manifestations appear. In particular, BCAA levels could represent a harbinger of metabolic risk even in individuals with normal insulin levels or homeostasis model assessment of insulin resistance (HOMA-IR) values within the reference limit (11).

Based on this background, we investigated the differences in plasma amino acid levels between children with and without obesity, as well as the variations in various amino acid indices. In addition, we examined the differences in amino acid profiles among children with obesity according to the presence or absence of insulin resistance. Furthermore, we evaluated the accuracy of amino acid-based software in precisely distinguishing children with obesity from controls without obesity.

## 2 Methods

This study enrolled 97 children aged 5–18 years, including 56 children diagnosed with obesity (study group) and 41 age- and sex-matched healthy children (control group). Obesity was defined as a BMI exceeding the 95th percentile based on national age-specific BMI percentile tables, while controls had BMI values between the 5th and 85th percentiles. All participants had no history of infection, chronic systemic disease, or prior use of any medications that could affect metabolic parameters. Blood samples were collected after an overnight fast, and participants who did not meet the fasting requirements were excluded from the study. Anthropometric measurements, including height and weight, were obtained using standard equipment with a precision of 0.1 cm and 0.1 kg, respectively. BMI was calculated and converted to standard deviation (SD) scores based on national growth references. The study protocol was approved by the Institutional Ethics Committee (Approval No: 2024/22/991) and conducted in accordance with the principles of the Declaration of Helsinki.

### 2.1 Sample collection

Serum and whole blood samples were drawn from each patient after 8–12 h of fasting for biochemical and amino acid analyses. Serum samples collected in serum separating tubes (Vacuette®, Greiner) were allowed to clot for 30 min and then centrifuged at 3,600 rpm for 15 min for routine chemistry and hormone analysis. Serum samples were analyzed on the same day as part of routine analysis. Whole blood samples collected into potassium ethylenediaminetetraacetic acid-containing tubes were used for HbA1C analysis. To perform amino acid analysis, whole blood samples were collected in potassium EDTA (K_2_EDTA)-containing tubes (Vacuette®, Greiner) and centrifuged at 4,000 rpm for 5 min to obtain plasma samples. Plasma samples were frozen at −20°C and measured in the following 2–3 days.

### 2.2 Biochemical analysis

Commercial kits were used for all chemistry and hormone parameters, including fasting plasma glucose, triglyceride (TG), and lipids (total cholesterol, HDL-C, LDL-C), aspartate aminotransferase (AST), alanine aminotransferase (ALT), and uric acid (UA) (Beckman Coulter, USA). Serum levels of glucose, TG, AST, ALT, and UA were measured in a biochemistry autoanalyzer (AU5800 Series Clinical Chemistry Analyzer, Beckman Coulter, USA). The HbA1C analysis was performed on the same day in boronate affinity chromatography (Premier Hb9210, Trinity Biotech, Ireland), and a commercially available kit of the same manufacturer was used. Serum insulin levels were determined in a hormone autoanalyzer (UniCel DxI 800 Access Immunoassay System, Beckman Coulter, USA).

For plasma concentrations of 44 amino acids, the experiments were performed on Agilent high-performance liquid chromatography (HPLC) system (Agilent Technologies, Santa Clara, CA, USA) consisting of flexible pump (G7104A), column compartment (G7116B), and autosampler (G7129C) coupled to Agilent Ultivo Triple Quadrupole LC–MS (6465B, Agilent Technologies, Santa Clara, CA, USA) equipped with electrospray ionization source (ESI). For the determination of the concentrations of the underivatized free amino acids, JASEM Amino Acids LC–MS/MS analysis kit (product number JSM-CL-500) was used (Altium Laboratuvar Cihazları. AŞ, İstanbul, Türkiye). The analysis of the underivatized free amino acids was performed using calibration standards to create calibration curves, mobile phases (mobile phase A and B), an analytical column tailored for simultaneous analysis of the compounds, and chromatographic and mass detection parameters of the analytical method. The HPLC system was operated to inject 3 μL of treated calibrators/samples into the analytical column which was maintained at 30°C. The temperature of the autosampler was kept at 8°C. The chromatographic separation was performed utilizing mobile phases A and B with gradient elution at a flow rate of 0.7 mL/min. The HPLC elution was performed as follows: the initial LC gradient of 22% A was held for 1 min. Subsequently, the gradient was increased linearly to 78% B within 3.0 min and maintained for 0.5 min. Finally, the column was equilibrated at 22% A for 3 min. The total running time was 7.5 min. Mass detections of amino acids were conducted in positive ion multiple reaction monitoring mode. The mass spectrometer settings of the analytical method were as follows: drying gas temperature 150 °C, drying gas flow 10 L/min, nebulizer pressure 40 psi, sheath gas temperature 400 °C, sheath gas flow 10 L/min, and capillary voltage 2,000 V. The MS/MS detections were achieved by product ion transitions generated by collision-induced dissociation (CID) of the corresponding precursor ion. Plasma amino acids' limit of quantification (LOQ) values and linearity ranges are provided in Supplementary Table S1.

Amino acid-based metabolic risk indices were calculated using Amino-Check® software (Amino Acid Science Ltd., London, UK), which integrates specific amino acid ratios and concentrations based on previously validated multivariate models, including visceral adiposity indices defined by Yamakado et al. (12), insulin resistance, cardiovascular disease, type 2 diabetes, and metabolic dysfunction-associated steatotic liver disease (MASLD). Parameters such as the glutamic acid/glutamine, BCAA-related, and essential/non-essential amino acid (EAA/NEAA) ratios were included in the stratification algorithms defined by Amino Acid Science Ltd. Details of the measured individual plasma amino acids and metabolic risk indices are presented in Supplementary Table S2.

### 2.3 Data visualization analyses

To plot the overall metabolic variations among groups and find significantly changed amino acids, a volcano plot and principal component analysis (PCA) were used. PCA was used to evaluate the general difference in plasma amino acid profiles between the obesity and control groups, and 95% confidence ellipses were plotted to visualize clustering patterns among groups. The first two principal components were used, capturing the maximum variation within the amino acid dataset. Volcano plot analysis was used to simultaneously display the statistical significance (*p*-values) and biological significance (fold changes) of individual amino acids between groups. The volcano plot was constructed by plotting the negative log10 of the *p*-values (*y*-axis) against the log_2_ fold change values (*x*-axis) for each amino acid. Statistical significance thresholds were set at *p* < 0.05, and fold change thresholds were established to identify amino acids with both statistical and biological significance. Significantly altered amino acids were highlighted and labeled on the plot to facilitate interpretation of metabolic differences between children with obesity and healthy controls (Figure 2 and Supplementary Figure S1).

### 2.4 Statistical analysis

The normality of the quantitative variables was assessed using skewness and kurtosis, with values within ±2 indicating a normal distribution. Normally distributed variables were presented as the mean ± SD and compared between the groups using the independent-samples *t*-test and one-way analysis of variance (ANOVA). Non-normally distributed variables were summarized as mean ranks and analyzed using the Mann–Whitney *U*-test and Kruskal–Wallis *H*-test. Categorical variables were expressed as frequencies and percentages, and group comparisons were performed using the chi-squared test. To investigate the relationships between quantitative variables, Pearson's correlation coefficient was used for normally distributed data, whereas Spearman's correlation coefficient was applied for non-normally distributed variables. Receiver operating characteristic (ROC) curve analysis was used to evaluate the diagnostic performance of the selected parameters, and the area under the ROC curve (AUC) was reported as an indicator of model efficiency.

All statistical analyses were performed using IBM SPSS Statistics version 25.0 (IBM Corp., Armonk, NY, USA), and a two-tailed *p* < 0.05 was considered statistically significant.

## 3 Results

### 3.1 Group characteristics and anthropometric findings

**Figure 1:**
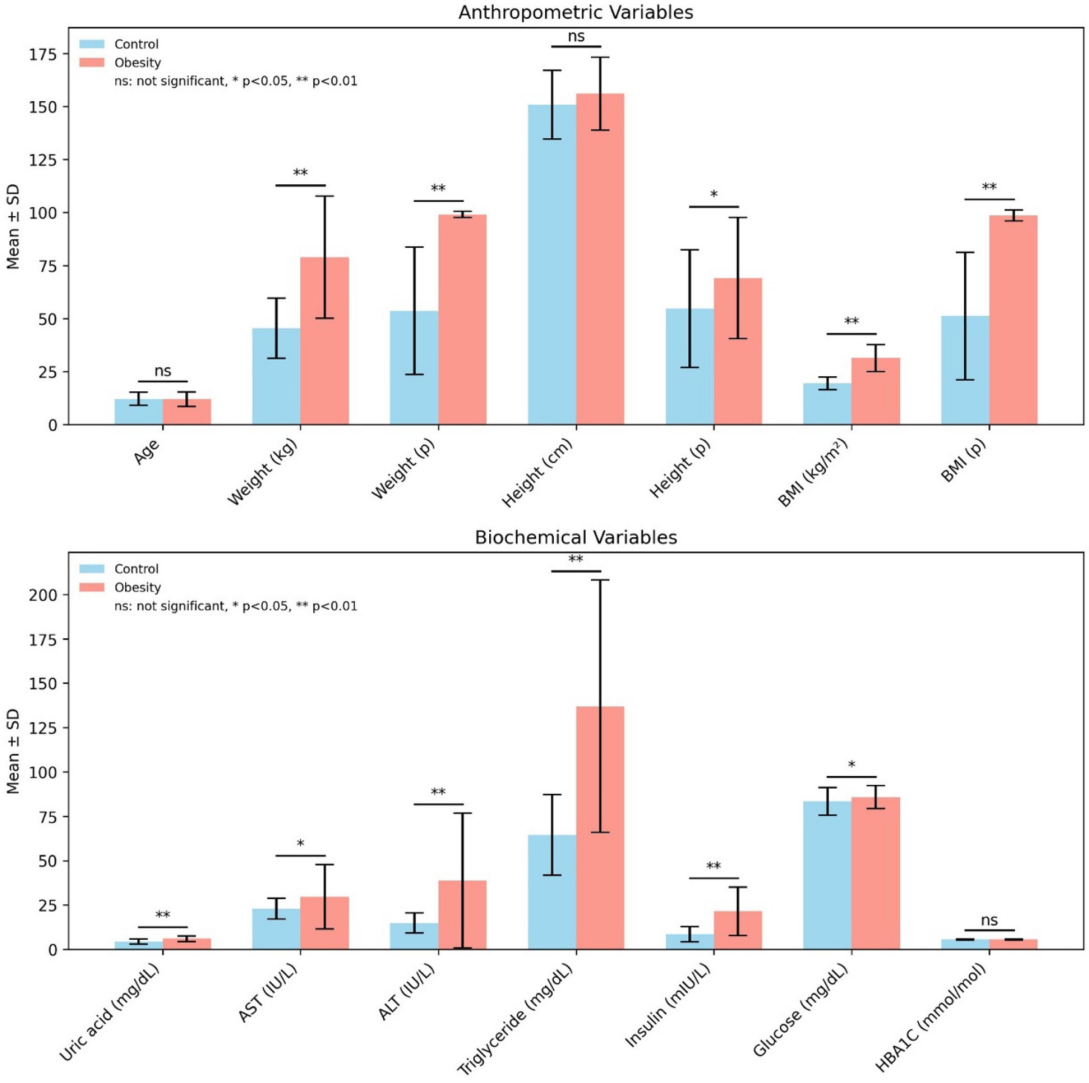
Anthropometric and biochemical variables in the control and obesity groups. Data are presented as mean ± standard deviation (SD). For normally distributed variables, comparisons between groups were performed using the independent sample *t*-test, whereas for non-normally distributed variables, the Mann–Whitney *U*-test was applied. Statistical significance is indicated as *p < 0.05; **p < 0.01 and; ns, not significant. BMI, body mass index; AST, aspartate aminotransferase; ALT, alanine aminotransferase; HbA1c, hemoglobin A1c.

The mean age of the included children was 11.98 ± 3.45 years in children with obesity vs. 12.12 ± 3.12 years in the control group (*p* = 0.838). Children with obesity had a male predominance (57.1%), whereas the control group included a higher proportion of females (61.0%). However, the sex distribution did not differ significantly between the groups (*p* = 0.119). Similarly, the age distribution (5–8, 9–13, 14–18) was comparable between the three groups (*p* = 0.858). Children with obesity had significantly higher mean body weight (78.94 ± 28.82 kg vs. 45.42 ± 14.21 kg, *p* < 0.001), weight percentile (99.10 ± 1.41 vs. 53.67 ± 30.06, *p* < 0.001), and BMI percentile (98.64 ± 2.53 vs. 51.14 ± 30.02, *p* < 0.001) than those in the control group. Although height did not significantly differ between the groups (*p* = 0.133), the height percentile was significantly higher in children with obesity (*p* = 0.015, Figure 1).

### 3.2 Biochemical and metabolic parameters

Fasting insulin levels were markedly higher in children with obesity compared with those in the control group (21.51 ± 13.69 IU/mL vs. 8.53 ± 4.25 µIU/mL, *p* < 0.001). Although fasting glucose levels were slightly higher in children with obesity (85.82 ± 6.49 mg/dL) compared with those in the control group (83.44 ± 7.78 mg/dL), the difference reached statistical significance (*p* = 0.033) but was clinically modest. Serum ALT (38.86 ± 38 U/L vs. 14.80 ± 5.67 U/L, *p* < 0.001) and AST (29.66 ± 18.16 U/L vs. 22.90 ± 5.84 U/L, *p* = 0.04) levels were significantly higher in children with obesity. TG levels were also significantly increased in children with obesity (136.98 ± 71.08 mg/dL vs. 64.54 ± 22.83 mg/dL, *p* < 0.05). Although HDL cholesterol levels were lower in children with obesity, the difference did not reach statistical significance. UA concentrations were significantly higher among children with obesity compared with those in controls (6.01 ± 1.57 mg/dL vs. 4.46 ± 1.44 mg/dL, *p* < 0.01), supporting its association with obesity-related metabolic stress (Figure 1).

### 3.3 Alterations in plasma amino acid profiles

**Figure 2:**
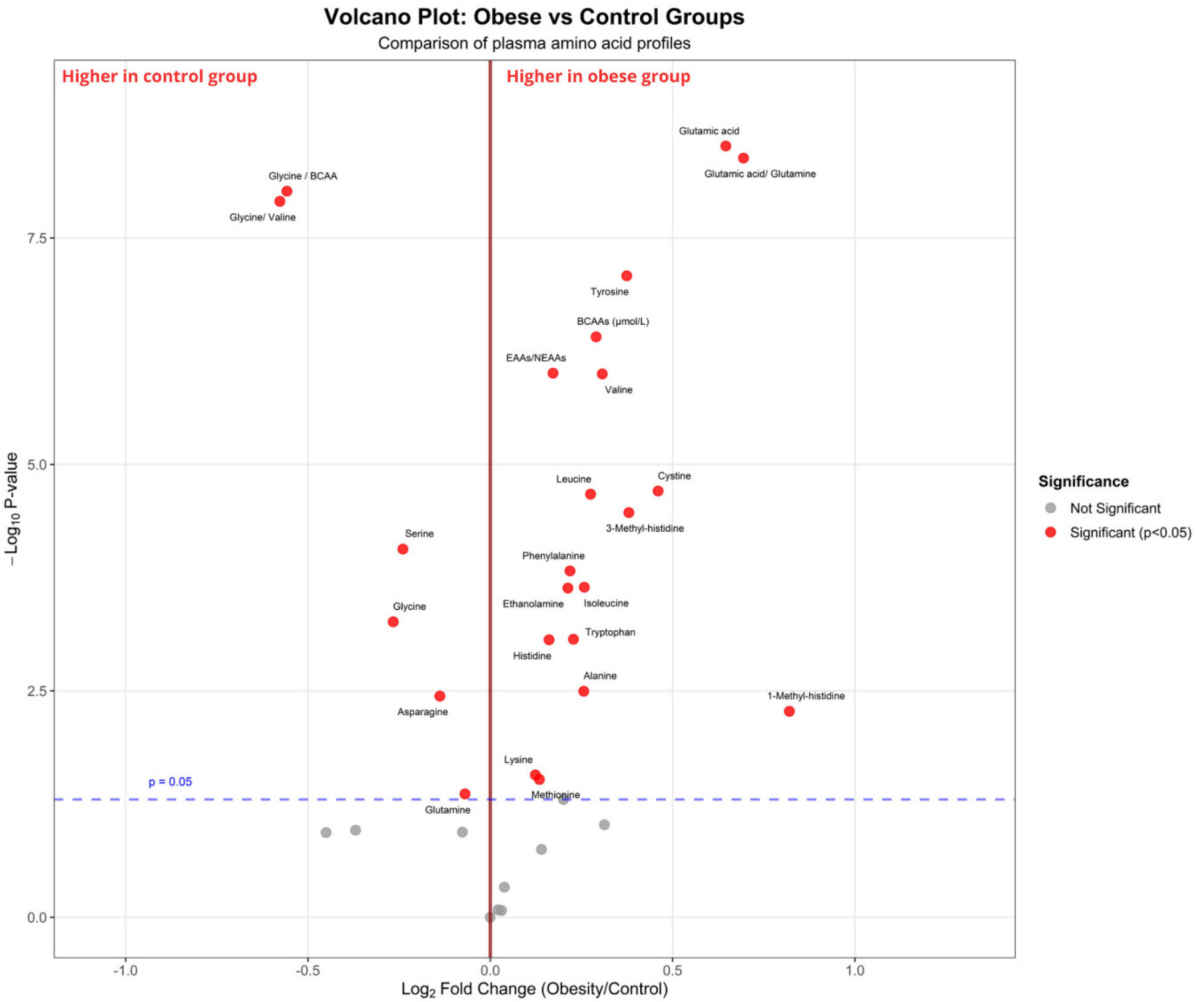
Volcano plot comparing plasma amino acid profiles between children with obesity and control groups. The *x*-axis represents the log_2_ fold change (children with obesity vs. control), and the *y*-axis represents the –log_10_ *p*-value from statistical testing. The vertical red line indicates no fold change (log_2_FC = 0), while the horizontal dashed blue line corresponds to the significance threshold (*p* = 0.05). The red dots indicate metabolites that are significantly different between groups (*p* < 0.05), and the gray dots represent non-significant metabolites. Amino acids enriched in the group with obesity [e.g., glutamic acid, tyrosine, branched-chain amino acids (valine, leucine, isoleucine)] are shown on the right, whereas those enriched in the control group (e.g., glycine, serine, glycine/BCAA ratio) are shown on the left.

**Figure 3:**
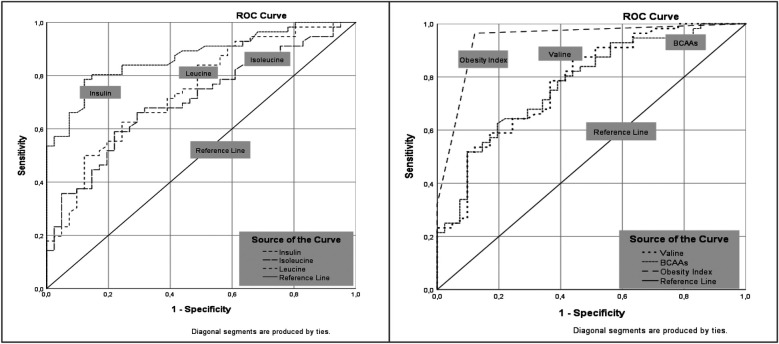
ROC curves showing the discriminatory performance of insulin, isoleucine, leucine, valine, total branched-chain amino acids (BCAAs), and the obesity index in distinguishing children with obesity from controls. The area under the curve (AUC) values were calculated for each parameter, with higher AUC values indicating greater discriminatory ability.

The concentrations of BCAAs were significantly higher in children with obesity than those in the control group (*p* < 0.001). The levels of other essential and non-essential amino acids, such as phenylalanine, histidine, tyrosine, alanine, glutamic acid, 1-methyl-histidine, 3-methyl-histidine, and ethanolamine, were also significantly elevated in children with obesity (all *p* < 0.05). Conversely, glycine, serine, and asparagine levels were significantly lower in children with obesity than those in the control group (all *p* < 0.001). Furthermore, glycine/BCAA, glycine/valine, and glutamic acid/glutamine ratios were significantly altered in children with obesity, indicating early metabolic disorders (all *p* < 0.05, Figures 2, 3 and Supplementary Table S3).

### 3.4 Diagnostic performance of biomarkers

ROC curve analyses demonstrated the significant diagnostic potential of several biomarkers for distinguishing children with obesity from healthy controls. Fasting insulin exhibited the highest predictive power among the individual biomarkers, with an AUC of 0.87 and a cutoff of 13.15 µIU/mL, which yielded a sensitivity and specificity of 79% and 88%, respectively; Figure 3).

Regarding amino acid biomarkers, valine (AUC = 0.78, cutoff = 254.80 µmol/L), leucine (AUC = 0.74, cutoff = 138.85 µmol/L), and isoleucine (AUC = 0.71, cutoff = 83.65 µmol/L) demonstrated moderate diagnostic accuracy. The combined BCAA level yielded an AUC of 0.78 with a sensitivity of 63% and specificity of 81% at a cutoff of 502.95 µmol/L. The Amino-Check®-derived obesity risk index demonstrated the highest overall discriminatory power (AUC = 0.94, cutoff = 1.50, sensitivity = 96%, specificity = 88%).

All ROC analyses demonstrated statistical significance (*p* < 0.05), supporting the potential use of targeted amino acid profiling, specifically BCAAs and amino acid-based composite indices, for the early diagnosis and stratification of metabolic risk in pediatric obesity.

### 3.5 Associations between clinical variables

According to the results of the analysis, there were no significant differences between children with obesity and control groups concerning sex and age (both *p* > 0.05); however, children with obesity included higher proportions of individuals with insulin, isoleucine, leucine, valine, BCAA, tryptophan, phenylalanine, and tyrosine levels above the cutoff than those in the control group (all *p* < 0.05, Table 1).

**Table 1:** Comparison of the control and obesity groups using defined cutoff values for specific anthropometric, biochemical, and amino acid-related variables.

Parameters	Control (*n* = 41)		Obesity (*n* = 56)		Total		*p*
	Number	%	Number	%	Number	%	
Sex							
Female	25	61.0	24	42.9	49	50.5	0.12
Male	16	39.0	32	57.1	48	49.5	
Age (years)							
5–8	8	19.5	10	17.9	18	18.6	0.86
9–13	16	39.0	25	44.6	41	42.3	
14–18	17	41.5	21	37.5	38	39.2	
Insulin (mIU/L)							
<13.15	36	87.8	12	21.4	48	49.5	0.00*
≥13.15	5	12.2	44	78.6	49	50.5	
Isoleucine (µmol/L)							
<83.65	32	78.0	23	41.1	55	56.7	0.00*
≥83.65	9	22.0	33	58.9	42	43.3	
Leucine (µmol/L)							
<138.85	31	75.6	21	37.5	52	53.6	0.00*
≥138.85	10	24.4	35	62.5	45	46.4	
Valine (µmol/L)							
<254.80	23	56.1	7	12.5	30	30.9	0.00*
≥254.80	18	43.9	49	87.5	67	69.1	
BCAAs (µmol/L)							
<502.95	33	80.5	21	37.5	54	55.7	0.00*
≥502.95	8	19.5	35	62.5	43	44.3	
Tryptophan (µmol/L)							
<47.25	18	43.9	9	16.1	27	27.8	0.01*
≥47.25	23	56.1	47	83.9	70	72.2	
Phenylalanine (µmol/L)							
<55.80	28	68.3	17	30.4	45	46.4	0.00*
≥55.80	13	31.7	39	69.6	52	53.6	
Tyrosine (µmol/L)							
<72.05	30	73.2	11	19.6	41	42.3	0.00*
≥72.05	11	26.8	45	80.4	56	57.7	

*p*, chi-squared test of independence.

* *p* < 0.05.

Using the developed obesity risk index (>1, children with obesity; ≤1, children without obesity), the vast majority of children with obesity (96.4%) were accurately marked as a group with obesity, whereas only 12.2% of participants in the control group were considered children with obesity. The estimated sensitivity and specificity of the obesity risk index were 96% and 87%, respectively, with comparisons made between participants’ scores. In addition, the obesity risk index performed significantly better than BCAA levels in accurately identifying children at risk of obesity (96.4% vs. 62.5%).

**Figure 4:**
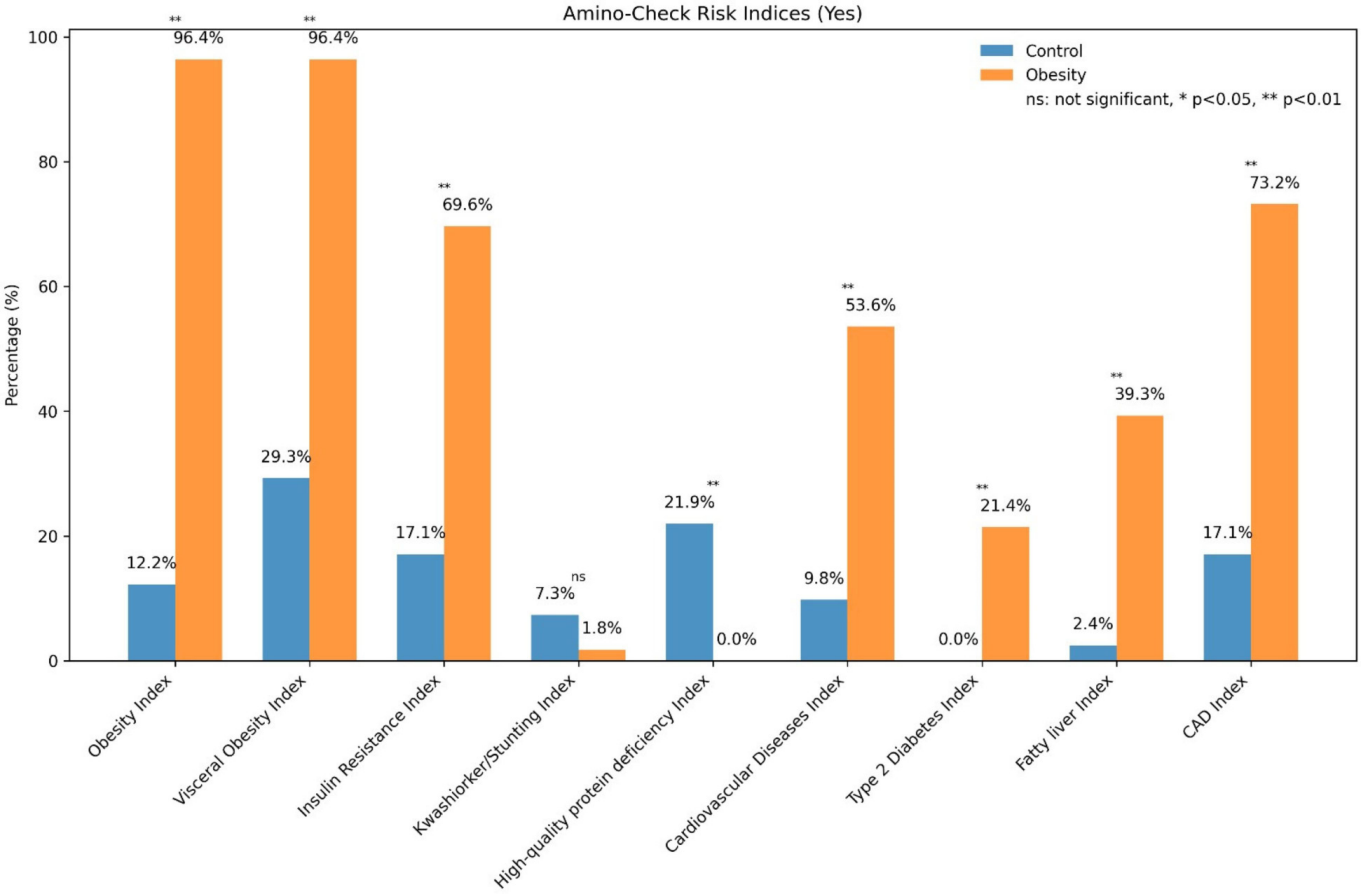
Comparison of Amino-Check®-derived metabolic risk indices between the control and obesity groups. The bars represent the percentage of individuals (%) with positive risk for each index. The indices include obesity index, visceral obesity, insulin resistance, cardiovascular disease risk, type 2 diabetes risk, MASLD (fatty liver) risk, coronary artery disease risk, high-quality protein deficiency, and kwashiorkor/stunting. Statistical comparisons were performed using the chi-square test. Significance levels are indicated as *p* < 0.05 (*), *p* < 0.01 (**), and ns, not significant.

The visceral obesity risk index was markedly higher in children with obesity (96.4 ± 5.8%) compared with findings in the control group (58.06 ± 8.2%). The insulin resistance risk index indicated that 69.6 ± 7.1% of children with obesity and 17.1 ± 4.6% of controls were at risk of developing insulin resistance. The cardiovascular disease risk index illustrated that the elevated risk of developing cardiovascular diseases in children with obesity (53.6 ± 6.5%) compared with the control group (9.8 ± 3.2%). The type 2 diabetes risk index revealed that 21.4 ± 4.9% of children with obesity were at risk of diabetes, compared with none in the control group. Finally, the MASLD risk index was 39.3 ± 6.0% in children with obesity compared with 2.4 ± 1.2% in the control group (Figure 4).

To evaluate the effect of adolescence on metabolic markers, children with obesity were divided into three age groups: 5–8, 9–13, and 14–18 years. No statistically significant differences were observed between the three groups regarding BMI percentile, weight percentile, height percentile, and TG, fasting insulin, fasting glucose, and HBA1C levels (Tables 2, 3). The visceral fat area (VFA) index was significantly higher in adolescents, with the highest mean value observed in the 14–18-year group, followed by the 9–13-year group, and the lowest values in the 5–8-year group (*p* < 0.001). Total BCAA levels differed significantly across age groups (*p* < 0.001), with the highest values observed in the 14–18-year group, intermediate levels in the 9–13-year group, and the lowest levels in the 5–8-year group. In contrast, fasting insulin levels did not show a statistically significant difference among the three age groups (Table 4).

**Table 2:** Comparison of anthropometric measurements (weight, height, and BMI) among different age groups (5–8, 9–13, and 14–18 years) in children with obesity.

Anthropometric parameters	(a) 5–8 years, *n* = 10	(b) 9–13 years, *n* = 25	(c) 14–18 years, *n* = 21	*F*/*χ*^2^	*p* (difference)
	$\bar{x}\pm \mathrm{SD}$	$\bar{x}\pm \mathrm{SD}$	$\bar{x}\pm \mathrm{SD}$		
Weight (kg)	49.17 ± 17.28	68.46 ± 17.53	105.60 ± 21.33	F: 36.690	0.00* (a < b, c), (b < c)
Weight (percentile)	99.53 ± 0.69	98.77 ± 1.59	99.31 ± 1.40	*χ*^2^: 5.960	0.06
Height (cm)	134.50 ± 10.78	151.60 ± 11.36	171.67 ± 9.73	*F*: 45.038	0.00* (a < b, c), (b < c)
Height (percentile)	74.66 ± 29.40	75.72 ± 26.28	58.45 ± 29.01	*F*: 2.438	0.10
BMI (kg/m^2^)	26.58 ± 5.60	29.39 ± 4.29	35.96 ± 6.17	*F*: 13.747	0.00* (c > a, b)
BMI (percentile)	99.42 ± 0.53	98.16 ± 3.32	98.83 ± 1.91	*χ*^2^: 3.973	0.14

*χ*^2^, Kruskal–Wallis *H*-test; *F*, one-way ANOVA.

Data are presented as mean ± standard deviation (SD).

* *p* < 0.05.

**Table 3:** Comparison of biochemical parameters (uric acid, AST, ALT, triglyceride, insulin, glucose, HbA1c) among children with obesity in three age groups (5–8, 9–13, and 14–18 years).

Biochemical parameters	**(a) 5–8 years, *n* = 10**	**(b) 9–13 years, *n* = 25**	**(c) 14–18 years, *n* = 21**	***F*/*χ*^2^**	***p* (difference)**
	Mean ± SD	Mean ± SD	Mean ± SD		
Uric acid (mg/dL)	4.71 ± 0.91	5.71 ± 1.63	6.98 ± 1.11	*F*: 10.758	0.00* (c > a, b)
AST (IU/L)	31.00 ± 17.99	30.64 ± 22.05	27.86 ± 13.13	*χ*^2^: 0.193	0.91
ALT (IU/L)	30.10 ± 31.25	37.36 ± 41.58	44.81 ± 37.12	*χ*^2^: 4.187	0.12
Triglyceride (mg/dL)	95.70 ± 37.38	136.40 ± 66.00	157.33 ± 82.17	*F*: 2.706	0.08
Insulin (mIU/L)	19.94 ± 24.31	19.84 ± 7.39	24.24 ± 13.09	*χ*^2^: 4.194	0.12
Glucose (mg/dL)	85.40 ± 5.76	86.56 ± 5.82	85.14 ± 7.68	*F*: 0.290	0.75
HBA1C (mmol/mol)	5.65 ± 0.26	5.63 ± 0.25	5.56 ± 0.24	*F*: 0.537	0.59

*χ*^2^, Kruskal–Wallis *H*-test; *F*, one-way ANOVA.

Data are presented as mean ± standard deviation (SD).

* *p* < 0.05.

**Table 4:** Analysis of plasma amino acid levels among various age groups (5–8, 9–13, and 14–18 years) in children with obesity.

Amino acids (µmol/L)	(a) 5–8 years, *n* = 10	(b) 9–13 years, *n* = 25	(c) 14–18 years, *n* = 21	***F*/*χ*^2^**	*p* (difference)
	$\bar{x}\pm \mathrm{SD}$	$\bar{x}\pm \mathrm{SD}$	$\bar{x}\pm \mathrm{SD}$		
Lysine	159.43 ± 23.72	175.55 ± 19.39	194.28 ± 38.04	*F*: 5.545	0.01* (c > a)
Methionine	25.04 ± 3.08	25.88 ± 3.82	30.63 ± 5.78	*F*: 7.979	0.00* (c > a, b)
Tryptophan	51.97 ± 10.37	56.05 ± 9.43	63.72 ± 11.62	*F*: 5.229	0.01* (c > a)
Isoleucine	72.58 ± 15.18	81.72 ± 14.91	102.77 ± 19.30	*F*: 14.165	0.00* (c > a, b)
Leucine	130.82 ± 22.73	140.34 ± 18.74	169.15 ± 30.03	*F*: 11.665	0.00* (c > a, b)
Valine	274.00 ± 43.76	299.89 ± 38.85	352.38 ± 73.17	*F*: 8.561	0.00* (c > a, b)
Phenylalanine	58.53 ± 11.86	57.97 ± 6.75	68.12 ± 14.76	*χ*^2^: 10.097	0.01* (c > b)
Histidine	81.69 ± 9.06	89.13 ± 12.14	93.95 ± 11.56	*F*: 3.924	0.03* (c > a)
Threonine	129.69 ± 27.50	133.29 ± 27.99	140.47 ± 28.73	*F*: 0.615	0.55
Tyrosine	80.83 ± 12.05	87.11 ± 13.69	85.13 ± 19.90	*F*: 0.545	0.58
Glutamine	528.14 ± 48.12	548.11 ± 70.82	575.48 ± 70.94	*F*: 1.885	0.16
Glycine	206.35 ± 45.48	203.94 ± 56.33	239.27 ± 58.23	*F*: 2.573	0.09
Serine	114.73 ± 25.02	119.32 ± 19.98	119.11 ± 23.15	*F*: 0.170	0.84
Cysteine	24.77 ± 12.35	26.17 ± 6.05	28.19 ± 8.77	*F*: 0.634	0.53
Proline	159.63 ± 33.75	201.11 ± 54.18	262.30 ± 92.79	*χ*^2^: 15.320	0.00* (c > a, b)
Arginine	45.97 ± 18.73	47.94 ± 16.52	54.68 ± 21.84	*χ*^2^: 3.170	0.21
Citrulline	25.73 ± 7.46	24.56 ± 5.31	27.21 ± 6.61	*F*: 1.041	0.36
Ornithine	82.80 ± 14.50	102.86 ± 26.75	106.76 ± 33.16	*F*: 2.641	0.08
Taurine	44.79 ± 15.80	43.66 ± 10.32	45.21 ± 14.13	*χ*^2^: 0.092	0.96
Alanine	371.49 ± 100.34	425.71 ± 83.93	501.30 ± 118.44	*F*: 6.371	0.01* (c > a)
Asparagine	43.42 ± 5.72	47.10 ± 8.63	52.96 ± 11.03	*F*: 4.247	0.02* (c > a)
Aspartic acid	5.24 ± 1.15	6.09 ± 2.07	6.21 ± 1.67	*F*: 1.072	0.35
Glutamic acid	52.74 ± 13.69	46.78 ± 11.31	66.22 ± 23.78	*χ*^2^: 10.711	0.01* (c > b)
β-Alanine	2.72 ± 0.88	4.09 ± 2.11	4.77 ± 2.55	*χ*^2^: 7.421	0.02* (c > a)
OH-lysine	1.39 ± 0.67	1.56 ± 0.81	1.15 ± 0.59	*F*: 1.892	0.16
OH-proline	13.65 ± 2.48	15.92 ± 4.72	14.38 ± 6.06	*χ*^2^: 2.996	0.22
1-Methyl-histidine	6.72 ± 5.66	3.70 ± 3.99	5.83 ± 4.93	*F*: 1.985	0.15
3-Methyl-histidine	1.73 ± 0.57	1.64 ± 0.36	2.20 ± 0.51	*F*: 9.213	0.00* (c > b)
Ethanolamine	8.22 ± 1.26	8.56 ± 1.23	10.05 ± 1.77	*F*: 7.591	0.00* (c > a, b)
EAAs/NEAAs	0.61 ± 0.07	0.62 ± 0.06	0.62 ± 0.07	*F*: 0.135	0.87
	0.44 ± 0.09	0.40 ± 0.12	0.39 ± 0.11	*F*: 0.636	0.53
Glycine/valine	0.76 ± 0.15	0.69 ± 0.20	0.70 ± 0.20	*F*: 0.484	0.62
BCAAs (µmol/L)	477.40 ± 70.89	521.94 ± 66.21	624.30 ± 113.22	*F*: 12.245	0.00* (c > a, b)
VFA	1.33 ± 0.75	1.58 ± 0.82	2.68 ± 1.62	*χ*^2^: 9.292	0.01* (c > a, b)
Glutamic acid/glutamine	0.10 ± 0.03	0.09 ± 0.02	0.12 ± 0.05	*χ*^2^: 7.467	0.02* (c > b)

*χ*^2^, Kruskal–Wallis *H*-test; F, one-way ANOVA.

Data are given as mean ± standard deviation (SD) in µmol/L.

* *p* < 0.05.

## 4 Discussion

This study has provided novel insights into the metabolic signatures associated with pediatric obesity by evaluating plasma amino acid profiles and related indices. These findings underscore the potential utility of amino acid profiling in early risk stratification and metabolic phenotyping in pediatric populations. When placed within the current literature, our data suggest that altered amino acid metabolism could represent an early biomarker of cardiometabolic dysfunction in children, possibly occurring before the onset of overt insulin resistance or clinical metabolic syndrome.

Previous clinical studies consistently reported significant differences in BCAA and aromatic amino acid (AAA) levels between children with and without obesity (4, 13–17), similar to those observed in the present study. In addition, consistent with earlier research, we observed elevated glutamic acid levels (15, 17), a higher glutamic acid/glutamine ratio (18), and increased alanine concentrations (11, 19) in the group with obesity. Conversely, glycine levels were significantly lower in children with obesity, supporting previous reports (19, 20). Our findings also align with those of Bugajska et al., who reported that serine and asparagine levels were significantly reduced in the obesity group (11). Similarly, a recent publication by Campos et al. (20) highlighted increased BCAA and decreased glycine levels in overweight children, mirroring trends observed in adult populations. Our study replicated these patterns, further reinforcing the relevance of plasma amino acid profiling in identifying early metabolic risk in pediatric populations.

Previous studies frequently investigated the glutamic acid/glutamine ratio and suggested its potential as a biomarker of insulin resistance in adolescents with obesity (18, 21). In addition, this ratio is negatively associated with β-cell function in young individuals with type 2 diabetes (21). In our study, this ratio was significantly higher in the obesity group than that in the control group and was additionally elevated in subgroups with high fasting insulin levels (≥13.15 μIU/mL vs. <13.15 μIU/mL) and high BCAA concentrations (≥502.95 μmol/L vs. <502.95 μmol/L). These findings support previous reports and suggest that the glutamic acid/glutamine ratio is a valuable marker for assessing early metabolic risk assessment in children and adolescents.

We also found that 1-methyl-histidine and 3-methyl-histidine levels were significantly higher in the obesity group than those in the control group, consistent with the findings of Cosentino et al. (19). These amino acid derivatives, which are often associated with muscle protein turnover and dietary protein intake, could reflect altered metabolic states in obesity. Moreover, glycine/BCAA, glycine/valine, and glutamic acid/glutamine ratios were markedly disrupted in the obesity group. These altered ratios could provide additional insight into early metabolic dysregulation, particularly in cases in which total BCAA levels do not strikingly differ. Their use could enhance the early detection and risk stratification of metabolic disorders in pediatric populations.

Although several recent studies supported the role of BCAAs in obesity-related metabolic dysfunction and frequently reported elevated BCAA levels in children with obesity, some inconsistencies remain in the literature. Notably, two cross-sectional studies involving adolescents (22, 23) did not find significantly higher BCAA concentrations in children with obesity than in their normal-weight counterparts. These discrepancies highlight the importance of examining a broader spectrum of metabolic markers. Beyond individual BCAA levels, amino acid ratios and derivative indices could represent more sensitive indicators of early metabolic dysregulation. Recognizing this, our study included such metrics to detect meaningful differences between children with obesity and between metabolically distinct subgroups.

To this end, we used Amino-Check, an amino acid profiling and evaluation software developed by Amino Acid Science Ltd. This tool calculates several clinically relevant amino acid ratios and synthesizes them into a cardiometabolic risk stratification framework. The software integrates known amino acid biomarker patterns to generate indices related to visceral adiposity, insulin resistance, cardiovascular disease risk, type 2 diabetes susceptibility, and fatty liver potential. This personalized profiling approach could enhance early detection and facilitate more targeted preventive strategies in at-risk pediatric populations.

Although total BCAA levels were significantly higher in the obesity group than those in controls in our study, only 62.5% of children in the obesity group were correctly classified using a BCAA cutoff of 502.95 µmol/L. Interestingly, 19.5% of participants without obesity exceeded the cutoff. These findings underscore the limitation of relying solely on BCAA levels for distinguishing metabolic risk. To improve diagnostic precision, we integrated additional biomarkers, including glycine, tyrosine, alanine, 3-methyl-histidine, ornithine, valine, and the VFA index (VFI), using the Amino-Check risk scales. This composite model markedly improved obesity detection, increasing the correct classification rate from 62.5% to 91.5%. Such multivariate approaches could better capture the complexity of metabolic disturbances related to obesity.

Regional fat distribution, particularly visceral adiposity, is a well-established predictor of metabolic risk that surpasses BMI and total fat mass in clinical relevance. Visceral fat contributes to systemic inflammation and insulin resistance, increasing the risk of type 2 diabetes mellitus and cardiovascular disease (24). Furthermore, metabolomic signatures associated with visceral fat, including elevated BCAA, AAA, alanine, glycine, proline, glutamate, and tyrosine levels, have been linked to future type 2 diabetes and myocardial infarction, even among normal-weight individuals and adolescents (12, 25). To the best of our knowledge, this is the first study to evaluate the VFI in adolescents, extending the work of Yamakado et al. (12) in Japanese adults with obesity. In our study, the VFI was significantly higher in the obesity group than in the control group. This increase in visceral fat was paralleled by significantly higher fasting insulin and BCAA levels, reflecting the expected metabolic burden in pediatric obesity.

In this study, individuals with elevated BCAA levels exhibited significantly higher insulin levels and a higher VFA index. Similarly, the VFI and BCAA concentrations differed between groups stratified by fasting insulin levels. These findings indicate close associations of visceral fat accumulation with elevated insulin and BCAA levels. However, considering the dynamic hormonal changes during this developmental period, it is important to account for the potential confounding effects of puberty, as the surge in growth hormone and insulin-like growth factor-1 levels during puberty increases insulin resistance (26).

Although we observed no differences between the preadolescent and adolescent groups concerning BMI percentile, weight percentile, and fasting insulin, fasting glucose, and HbA1c levels, the VFI was significantly higher in the adolescent group, which likely reflects the effect of puberty on the fat distribution. More importantly, BCAA levels were significantly elevated in the adolescent subgroup, whereas insulin levels remained unchanged. These findings suggest that in pubertal children with obesity, elevated BCAA levels may occur independently of insulin levels or insulin resistance, and these changes are more directly correlated with visceral fat accumulation. This finding highlights a potentially unique metabolic signature in this age group and supports the role of BCAA profiling as a sensitive marker for visceral adiposity, particularly during adolescence.

Contrary to our findings, Zhang et al. (27) reported that BCAA and AAA levels were positively associated with insulin resistance during pubertal growth independent of adiposity. However, all participants in their study had normal BMI, and the methodology for assessing adiposity was not specified. Moreover, no data regarding adiposity levels were presented, which limits the interpretation of the independence claim. In another study (28), no significant differences in BCAA concentrations were observed between pubertal and prepubertal lean children despite the increase in insulin resistance during puberty. Similarly, another study of lean children found no link between BCAA levels and the pubertal status (29). This suggests that BCAA levels do not increase in lean adolescents despite insulin resistance associated with puberty. These discrepancies might be attributable to differences in the study populations. Unlike these studies, our research included children with obesity, in whom BCAA accumulation and altered amino acid metabolism might follow a different pathophysiological course, particularly in individuals with increased visceral adiposity. This emphasizes the need to consider the metabolic status when interpreting amino acid–puberty relationships.

Although insulin resistance commonly emerges during puberty (27), studies in healthy lean children revealed that this does not necessarily lead to elevated BCAA levels. This suggests that in cases in which BCAA concentrations increase during puberty, such increases might occur independently of insulin elevation, and they might represent early biomarkers of future cardiometabolic risk. Supporting this, McCormack et al. (13) demonstrated in a cross-sectional cohort of children and adolescents that elevated circulating BCAA levels were significantly associated with obesity and potentially predictive of the future development of insulin resistance.

Plasma amino acid profiling could represent a valuable tool for differentiating metabolically unhealthy obesity and metabolically healthy obesity (MHO) in children. Although clear associations of elevated BCAA and AAA levels with obesity were recorded in our cohort, a notable subset of children with obesity (37.5%) exhibited normal BCAA levels, and 21.4% had normal insulin concentrations. These children also displayed lower levels of ALT and lower visceral fat accumulation, supporting the presence of a more metabolically favorable profile despite their elevated BMI percentiles. These findings are consistent with the concept of MHO, a phenotype characterized by preserved insulin sensitivity and the absence of typical metabolic disturbances in the presence of excess adiposity (30, 31). Although the MHO phenotype is well documented in adults, with prevalence estimates ranging between 10% and 30%, its definition and long-term clinical implications in children remain debated (32, 33). Our findings suggest that plasma amino acid profiling, particularly BCAAs, could provide early metabolic evidence of MHO in pediatric populations, which could be critical for individualized risk stratification and prevention strategies. Notably, no significant sex-based differences in BCAA levels or insulin resistance indices were observed, suggesting that sex is not a major determinant of the observed metabolic variability. The heterogeneity observed among children with obesity underscores the need for a more nuanced approach beyond BMI alone that incorporates metabolic profiling to better understand underlying risk and tailor interventions accordingly.

Beyond the evaluation of BCAA and AAA levels, a comprehensive analysis of the entire plasma amino acid profile, including cardiometabolic risk assessment, enables a more nuanced risk stratification in children with obesity. Software database systems, such as Amino-Check, were developed using amino acid-based algorithms that integrate individual amino acid levels and their ratios to assess visceral obesity independently of general obesity. In addition, these systems might provide risk stratification tools for insulin resistance, cardiovascular disease, type 2 diabetes, and MASLD. In line with our findings, Bugajska et al. investigated early markers of cardiovascular disease and MASLD in overweight children with obesity. Their study revealed that abnormal amino acid profiles, together with elevated ALT and UA levels, were already present in prepubertal children, suggesting early metabolic disturbances that could predispose them to metabolic syndrome, MASLD, and increased cardiovascular risk (11). These findings support the utility of plasma amino acid profiling as a predictive and preventive metabolic screening tool.

In our study, ALT and UA levels were significantly elevated in the obesity group. The obesity risk index clearly demonstrated that insulin and fasting glucose levels, BCAA concentrations, and the VFA index, all of which correspond to statistically significant increases in cardiometabolic risks, were highest in children with high obesity scores. Moreover, a higher obesity risk index was consistently associated with greater severity of other cardiometabolic risk indicators, including visceral obesity, insulin resistance, type 2 diabetes, and coronary artery disease.

Although the levels of nutrients such as glucose, amino acids, and lipids are interconnected, amino acids play a critical role in obesity, as they reflect internal metabolic changes and the influence of the intestinal microbiota, acting as key metabolic modulators. Therefore, monitoring amino acids through metabolomic analysis could be a useful method for predicting metabolic disorders during childhood (7, 34).

One of the most important strengths of this study was its comprehensive evaluation of plasma amino acid profiles and their associations with metabolic risk indices in a pediatric population. Using validated stratification tools, such as the obesity risk index and VFA index, our study surpassed traditional anthropometric and biochemical markers to provide a metabolomic perspective on childhood obesity. In addition, the inclusion of preadolescent and adolescent subgroups provided a better understanding of the effects of development on amino acid metabolism. The use of high-sensitivity LC–MS/MS methods for amino acid quantification further enhanced the reliability and reproducibility of our biochemical data.

Despite these strengths, our study had several limitations. First, the cross-sectional and retrospective design limited our ability to identify the intercausal relationships between amino acid changes and metabolic outcomes. Second, dietary intake and physical activity, known factors regulating amino acid levels, were not quantitatively assessed, potentially introducing confounding effects. Third, although the sample size was sufficient for the main analyses, it might not have been sufficient to detect subtle sex-specific differences or interactions between biomarkers. Finally, the study was conducted at a single tertiary healthcare center, which might have limited the generalizability of the findings to a broader pediatric population. To further generalize our findings, longitudinal and multicenter studies incorporating lifestyle data and pubertal stages according to the Tanner criteria are required.

In conclusion, the routine use of amino acid profiling in children could help predict their future susceptibility to obesity, assess whether existing obesity represents a metabolic risk, and forecast insulin resistance and other cardiometabolic risks regardless of BMI. The development of software systems that use specific calculations for amino acids will provide opportunities to use more detailed amino acid profiles, and their integration with other metabolites will increase the predictive power of these assessments.

## Data Availability

The original contributions presented in the study are included in the article/Supplementary Material, further inquiries can be directed to the corresponding author.
