# Supplementary material for: Plasma amino acid profiles in pediatric obesity: potential biomarkers for the early assessment of metabolic risk
Source: Front Pediatr. 2025 Sep 26;13:1631302. doi: 10.3389/fped.2025.1631302 (PMC12512672; doi:10.3389/fped.2025.1631302)
Supplement: Supplementary file 1 [file Datasheet1.docx]

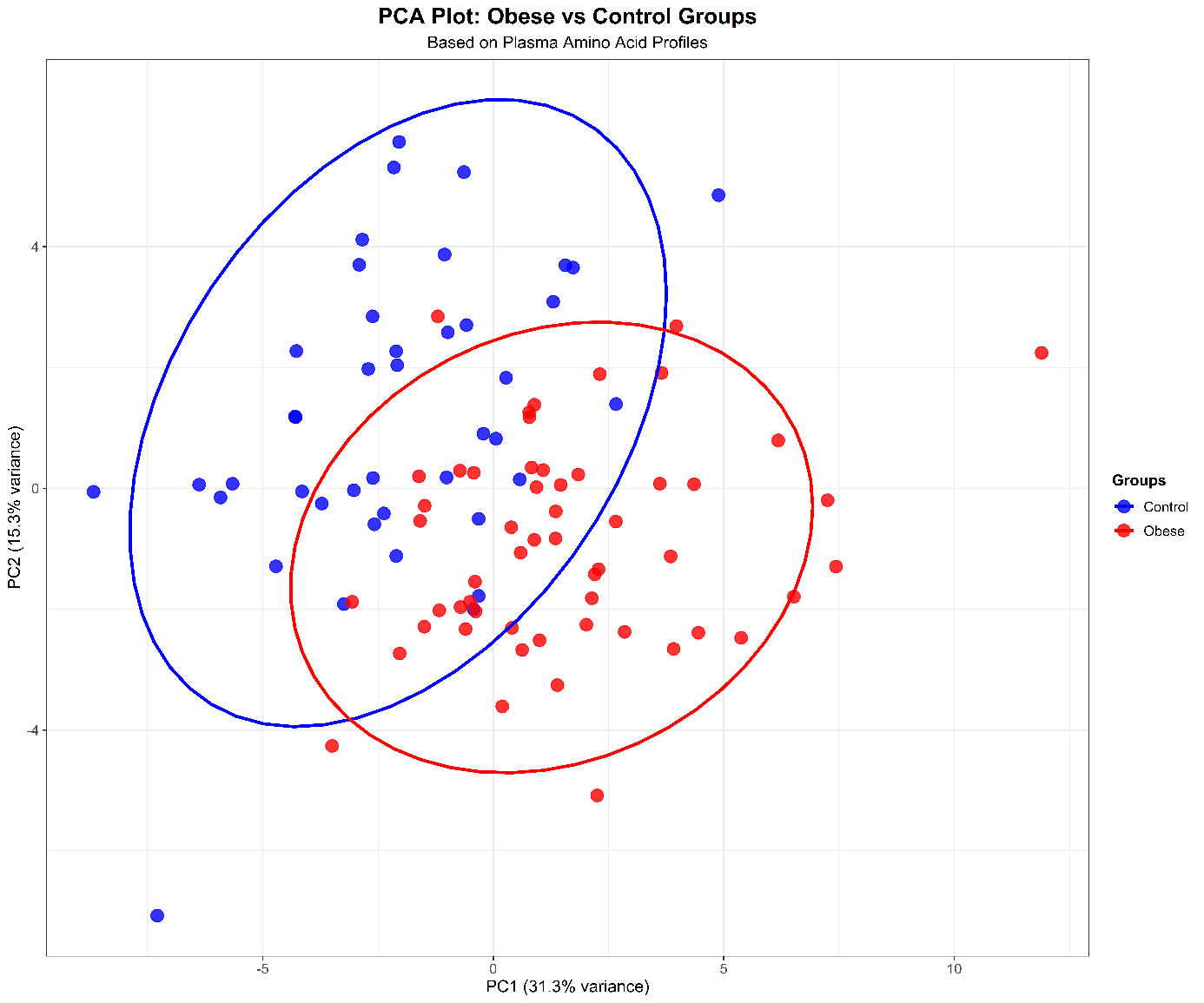


Supplementary Figure 1: PCA plot of the plasma amino acids in control vs. group with obesity. Each dot represents one participant (red = children with obesity, blue = control). PC1 (31.3% variance) and PC2 (15.3% variance) are shown. Ellipses denote 95% confidence intervals. Considerable overlap between groups indicates no distinct clustering.
