# Supplementary material for: Plasma amino acid profiles in pediatric obesity: potential biomarkers for the early assessment of metabolic risk
Source: Front Pediatr. 2025 Sep 26;13:1631302. doi: 10.3389/fped.2025.1631302 (PMC12512672; doi:10.3389/fped.2025.1631302)
Supplement: Supplementary file 2 [file Table1.docx]

Supplementary Material

Supplementary Table 1: Limit of Quantification (LOQ) values and linearity ranges for each plasma amino acid measured by LC-MS/MS. LOQ values are expressed in nmol/L and represent the lowest concentration of each amino acid that can be quantified with acceptable accuracy and precision according to the manufacturer’s validation protocol. Linearity indicates the analytical measurement range within which the assay response is directly proportional to the amino acid concentration. All values were obtained using the JASEM® commercial kit with the ESI Ultivo Triple Quadrupole LC/MS G6465 LC/QT system (Agilent Technologies, CA, USA).

|  | | LOQ* | Linearity |
| --- | --- | --- | --- |
|  |  | nmol/L | (R^2^ Correlation Coefficient) |
| 1 | Tryptophan | 0.23 | 0.9998 |
| 2 | Taurine | 5.47 | 0.9956 |
| 3 | Phenylalanine | 0.73 | 0.9970 |
| 4 | Tyrosine | 3.34 | 0.9991 |
| 5 | Leucine | 0.20 | 0.9997 |
| 6 | Isoleucine | 0.22 | 0.9991 |
| 7 | Methionine | 0.41 | 0.9999 |
| 8 | Gamma amino butyric acid | 0.07 | 0.9994 |
| 9 | 3-Amino isobutyric acid | 0.15 | 0.9995 |
| 10 | Norvaline | 0.15 | 0.9988 |
| 11 | L-2 aminoadipic acid | 0.83 | 0.9992 |
| 12 | ß-Alanine | 1.00 | 0.9990 |
| 13 | Ethanolamine | 0.56 | 0.9944 |
| 14 | Aspartic Acid | 1.06 | 0.9956 |
| 15 | 2-aminobutyric acid | 0.22 | 0.9998 |
| 16 | Threonine | 0.72 | 0.9999 |
| 17 | Serine | 0.89 | 0.9970 |
| 18 | Alanine | 6.82 | 0.9988 |
| 19 | Glycine | 9.03 | 0.9984 |
| 20 | Asparagine | 0.46 | 0.9997 |
| 21 | Trans-4-hydroxy-L-proline | 0.28 | 0.9914 |
| 22 | Glutamine | 0.32 | 0.9999 |
| 23 | Sarcosine | 4.38 | 0.9964 |
| 24 | Proline | 0.67 | 0.9998 |
| 25 | Homocitrulline | 0.89 | 0.9993 |
| 26 | Citrulline | 0.51 | 0.9977 |
| 27 | Homocystine | 0.31 | 0.9990 |
| 28 | Cystine | 0.97 | 0.9998 |
| 29 | Cystathionine | 0.25 | 0.9997 |
| 30 | Arginine | 0.15 | 0.9998 |
| 31 | Histidine | 2.43 | 0.9999 |
| 32 | Ornitine | 1.39 | 0.9996 |
| 33 | DL-5-Hydroxy lysine | 0.30 | 0.9988 |
| 34 | Lysine | 0.09 | 0.9988 |
| 35 | Carnosine | 0.12 | 0.9984 |
| 36 | 3-Methyl-L-Histidine | 0.30 | 0.9992 |
| 37 | 1-Methyl-L-Histidine | 0.20 | 0.9996 |
| 38 | Anserine | 0.21 | 0.9998 |
| 39 | Glutamic Acid | 0.24 | 0.9990 |
| 40 | Valine | 5.11 | 0.9986 |
| 41 | Ortho-Phospho-L-Serin | 0.12 | 0.9984 |
| 42 | Orth-Phosphoryl ethanolamine | 2.56 | 0.9976 |
| 43 | Arginino succinic acid | 0.62 | 0.9982 |
| 44 | Allo-isoleucine | 0.22 | 0.9998 |

Supplementary Table 2: List of plasma amino acids used in the calculation of Amino-Check™-based metabolic risk indices. The table includes all amino acids and relevant ratios incorporated into the algorithms for visceral fat area (VFA), insulin resistance (IR), cardiovascular disease (CVD), type 2 diabetes mellitus (T2DM), and metabolic dysfunction-associated steatotic liver disease (MASLD) risk estimation.

|  | **Description** |
| --- | --- |
| **Index / Ratio Name** | Ratio of total essential to non-essential amino acids |
| **Glycine / Branched-Chain Amino Acids** | Ratio of glycine to total BCAA (valine, leucine, isoleucine) |
| **Glycine / Valine** | Ratio of glycine to valine |
| **Glutamic Acid / Glutamine** | Ratio of glutamic acid to glutamine |
| **Visceral Fat Area (VFA) Index** | Multivariate index including glutamic acid, glycine, alanine, tyrosine, tryptophan, and BCAAs (Yamakado et al.) |
| **Obesity Risk Index** | Includes total BCAAs, glycine, tyrosine, alanine, 3-methyl-histidine, ornithine, valine, glutamic acid/glutamine ratio, and VFA Index |
| **Visceral Obesity Risk Index** | Includes total BCAAs and VFA Index |
| **Insulin Resistance Risk Index** | Includes total BCAAs, phenylalanine, tyrosine, alanine, alpha-aminobutyric acid, glutamic acid, and VFA Index |
| **Cardiovascular Disease Risk Index** | Includes total BCAAs, phenylalanine, tyrosine, arginine, citrulline, alpha-aminobutyric acid, glutamic acid, alanine, and VFA Index |
| **Type 2 Diabetes Risk Index** | Includes total BCAAs, alanine, glutamic acid/glutamine ratio, tyrosine, isoleucine, phenylalanine, glycine, glutamine, and VFA Index |
| **MASLD Risk Index** | Includes total BCAAs, VFA Index, glycine, serine, alanine, glutamic acid, threonine, tyrosine, phenylalanine |

Supplementary Table 3: Comparison of plasma amino acid profiles between the control and obesity groups. Data are expressed as mean ± standard deviation (µmol/L).

| Amino-acid (µmol/L) | Control  n=41 | Obesity  n=56 | T/Z | p |
| --- | --- | --- | --- | --- |
|  | ±SD | ±SD |  |  |
| Lysine | 167.30±30.80 | 179.69±30.76 | T: -1.960 | 0.06 |
| Methionine | 25.46±5.39 | 27.51±5.11 | T: -1.903 | 0.06 |
| Tryptophan | 50.50±10.41 | 58.20±11.24 | T: -3.438 | **0.00*** |
| Isoleucine | 74.48±14.48 | 87.98±20.36 | T: -3.624 | **0.00*** |
| Leucine | 124.25±24.83 | 149.44±28.54 | T: -4.532 | **0.00*** |
| Valine | 256.41±45.64 | 314.95±62.14 | T: -5.105 | **0.00*** |
| Phenylalanine | 53.78±16.15 | 61.88±12.08 | Z: -3.484 | **0.00*** |
| Histidine | 80.34±14.55 | 89.61±12.04 | T: -3.427 | **0.00*** |
| Threonine | 133.57±36.15 | 135.34±27.99 | T: -0.271 | 0.79 |
| Tyrosine | 66.76±15.47 | 85.25±15.96 | T: -5.707 | **0.00*** |
| Glutamine | 584.02±77.10 | 554.81±68.63 | T: 1.965 | 0.06 |
| Glycine | 262.09±68.83 | 217.62±56.95 | T: 3.477 | **0.00*** |
| Serine | 140.45±27.59 | 118.42±21.79 | T: 4.392 | **0.00*** |
| Cysteine | 19.55±7.93 | 26.68±8.40 | T: -4.224 | **0.00*** |
| Proline | 190.83±56.59 | 216.65±78.07 | Z: -1.709 | 0.09 |
| Arginine | 49.18±19.03 | 50.11±19.06 | T: -0.237 | 0.81 |
| Citrulline | 27.32±5.85 | 25.76±6.23 | T: 1.246 | 0.22 |
| Ornithine | 92.99±21.83 | 100.74±28.65 | Z: -0.971 | 0.33 |
| Taurine | 44.03±9.27 | 44.44±12.67 | Z: -0.332 | 0.74 |
| Alanine | 378.37±106.91 | 444.38±110.38 | T: -2.948 | **0.00*** |
| Asparagine | 53.75±10.34 | 48.64±9.76 | T: 2.484 | **0.02*** |
| Aspartic acid | 7.86±6.38 | 5.98±1.80 | Z: -1.717 | 0.09 |
| Glutamic acid | 35.06±9.70 | 55.13±19.29 | Z: -5.821 | **0.00*** |
| ß-alanine | 3.21±1.49 | 4.09±2.22 | Z: -1.848 | 0.07 |
| OH-lysine | 1.92±5.19 | 1.37±0.72 | Z: -1.463 | 0.14 |
| OH-proline | 15.30±6.41 | 14.94±5.00 | T: 0.312 | 0.76 |
| 1-Methyl-histidine | 2.91±3.38 | 5.04±4.75 | T: -2.442 | **0.02*** |
| 3-Methyl-histidine | 1.46±0.43 | 1.87±0.53 | T: -4.080 | **0.00*** |
| Ethanolamine | 7.90±1.87 | 9.05±1.63 | T: -3.172 | **0.00*** |
| EAAs/NEAAs | 0.55±0.07 | 0.62±0.07 | T: -4.978 | **0.00*** |
| Glycine/BCAA | 0.59±0.17 | 0.40±0.11 | T: 6.598 | **0.00*** |
| Glycine/valine | 1.05±0.31 | 0.71±0.19 | T: 6.642 | **0.00*** |
| BCAAs | 455.14±78.85 | 552.37±103.99 | T: -5.020 | **0.00*** |
| VFA | -0.07±0.79 | 1.95±1.29 | T: -8.839 | **0.00*** |
| Glutamic acid/glutamine | 0.06±0.02 | 0.10±0.04 | Z: -5.979 | **0.00*** |

Z: Mann–Whitney U test, T: Independent-samples *t*-test, *p < 0.05
